# 

**Published:** 1882-12

**Authors:** 


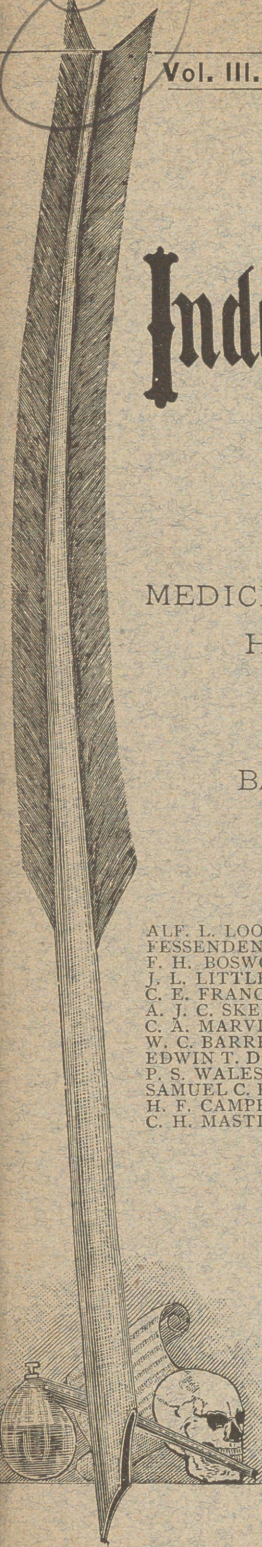

Vol. III. DECEMBER. 1882. No. 12.

S.G.O. INDEXED

THE

### Independent Practitioner:

### A MONTHLY JOURNAL,

DEVOTED TO

MEDICINE, SURGERY, OBSTETRICS, DENTISTRY, HYGIENE, AND POPULAR SCIENCE.

**EDITORIAL CORPS:**

BASIL M. WILKERSON, D.D.S., M.D., EDITOR.

**Associates:**

——— M.D. ——— D.D.S.

NEW YORK:
*THE INDEPENDENT PRACTITIONER CO.,*
VANDERBILT BUILDING,
132 NASSAU STREET.

**$3.00 Per Annum, in Advance. - - - Single Copies, 30 Cents.**

*Entered at the Post-Office, New York City, as Second-Class matter.*

